# An Illustration

**Published:** 1893-06

**Authors:** Howard Crutcher

**Affiliations:** Chicago


### AN ILLUSTRATION.

Howard Crutcher, M. D., Chicago.

A few weeks ago a dear old friend came to Chicago to pay me a social visit. Since last we met he has become a doctor of medicine, although he has not yet entered active practice.

He had not been around my home and office very long until he began to ask a great many questions. Without for a moment recognizing the fact that I knew him to be a mongrel, I preached Homœopathy to him and lost no chance to give the allies of allopathy masquerading in homœopathic ranks an unmerciful scoring. His superb intellectual equipment and his splendid literary attainments made me especially desirous of making a convert of him. A case of membraneous croup opened his eyes pretty wide, and some other cases caused him to readjust his glasses, but a personal experience completed the work.

One morning I entered his bed-room to look after his wants and found him suffering from terrible cramps and tenesmus. His constant desire to sit and strain, the nocturnal aggravation, the bloody mucous discharges, and the fetor of the breath left no doubt that *Mercurius-cor.* was his remedy.

His earnest request for a low potency was denied, as I do not recognize a patient's prejudice as having the least influence on the action of the high powers. A dose of the CM made such clean-cut work of the whole matter that his whole heart was won to Hahnemannian Homœopathy. He pleaded for a second dose, on the ground that "the pain might return," but I told him not to climb hills or to cross bridges until he reached them. Both the cure and the conversion were complete.

"Try it and publish the failures."

